# Clinical Utility of Repeat Sacroiliac Joint MRI in Patients Evaluated for Axial Spondyloarthritis: A Real-World Cohort Study

**DOI:** 10.3390/jcm15145363

**Published:** 2026-07-09

**Authors:** Sevilay Batıbay, Selin Cilli Hayıroğlu

**Affiliations:** Department of Rheumatology, Göztepe Prof. Dr. Süleyman Yalçın City Hospital, Istanbul Medeniyet University, Istanbul 34722, Türkiye; selincilli@hotmail.com.tr

**Keywords:** axial spondyloarthritis, sacroiliac joint, magnetic resonance imaging, repeat MRI, sacroiliitis, diagnostic reassessment, treatment modification

## Abstract

**Objectives**: To evaluate the real-world diagnostic and therapeutic impact of repeat sacroiliac joint magnetic resonance imaging (SIJ MRI) in patients undergoing assessment for axial spondyloarthritis (axSpA). **Methods**: This retrospective study included patients who underwent at least two SIJ MRI examinations from January 2010 to January 2026 at a single tertiary center. Demographic, clinical, laboratory, and imaging data were extracted from electronic medical records. MRI findings were classified according to Assessment of SpondyloArthritis International Society (ASAS) definitions. Changes between MRI1 and MRI2, diagnostic reassessment, treatment modification, and factors associated with diagnostic change were analyzed. **Results**: A total of 229 patients were included. The median interval between MRI examinations was 34 months. Among patients with initially negative or suspicious MRI findings, 13.0% converted to MRI-positive status on MRI2, whereas 44.8% of those with positive MRI1 findings regressed to negative or suspicious categories. Bone marrow edema (BME) was less frequent on MRI2 than MRI1 (38.0% vs. 50.2%, *p* = 0.003), while fat metaplasia (9.6% vs. 3.1%, *p* < 0.001) and sclerosis (34.9% vs. 26.6%, *p* = 0.006) were more common. Repeat MRI was associated with diagnostic reassessment in 41 patients (17.9%) and treatment modification in 27 patients (11.8%). In multivariate analysis, MRI1 BME positivity (OR 3.13, 95% CI 1.47–6.63, *p* = 0.003) and higher CRP levels (OR 1.06, 95% CI 1.01–1.11, *p* = 0.017) were independently associated with diagnostic reassessment. **Conclusions**: In routine clinical practice, repeat SIJ MRI was associated with diagnostic reassessment and treatment modification in a subset of patients undergoing evaluation for axSpA. Diagnostic reassessment was more frequently observed in patients with baseline inflammatory MRI findings and elevated inflammatory markers.

## 1. Introduction

Axial spondyloarthritis (axSpA) is a chronic inflammatory rheumatic disease characterized predominantly by involvement of the sacroiliac joints (SIJs) and spine. In the evaluation of axSpA, multiple clinical parameters contribute to the diagnostic process; however, imaging findings are frequently required to support the diagnosis [1]. The modified New York criteria (mNYC), based on conventional radiography, primarily assess structural damage and are insufficient for detecting early inflammatory changes, thereby contributing to diagnostic delay [2,3]. In contrast, magnetic resonance imaging (MRI) enables the visualization of active inflammatory lesions at an earlier stage and has become a cornerstone in the evaluation of patients with suspected non-radiographic axSpA (nr-axSpA) [4].

Despite the standardized definition of an “Assessment of SpondyloArthritis International Society (ASAS)-positive MRI” and subsequent refinements by the ASAS MRI working group [4], interpretation of SIJ MRI remains challenging in routine clinical practice. Imaging studies have identified several conditions that may mimic axSpA, including osteitis condensans ilii, degenerative or mechanical sacroiliac joint changes, and other degenerative spinal disorders [5]. Distinguishing early inflammatory disease from non-specific or mechanical MRI abnormalities therefore remains difficult in clinical practice [6], and this diagnostic uncertainty may lead clinicians to request repeat MRI examinations in selected patients.

Beyond diagnostic evaluation, previous studies and European Alliance of Associations for Rheumatology (EULAR) recommendations suggest that MRI-detected inflammatory activity may aid therapeutic decision-making in axial SpA, particularly when biologic treatment initiation or switching is being considered [6,7]. Recent real-world data further demonstrated that patients with active inflammatory findings on repeat MRI were more likely to undergo treatment escalation compared with those without active inflammation [8]. Current recommendations acknowledge that MRI may provide additional information for monitoring disease activity in axial SpA; however, they do not clearly define which patients should undergo repeat MRI or the optimal timing for repeat imaging [7]. Nevertheless, repeat MRI is often considered in clinical practice, particularly in patients with persistent symptoms or therapeutic uncertainty.

Previous studies have suggested that conversion from MRI-negative to MRI-positive status is relatively uncommon [9]. In contrast, the additional diagnostic yield of repeat imaging may be greater in human leukocyte antigen B27 (HLA-B27)-positive male patients [10]. Available evidence remains limited by small cohort sizes and short-term imaging outcomes, and the broader real-world impact of repeat MRI on diagnosis and treatment decisions has not been sufficiently explored.

Despite its widespread use, the clinical role of repeat MRI remains poorly defined. We therefore aimed to evaluate the real-world diagnostic and therapeutic contribution of repeat SIJ MRI in patients undergoing evaluation for axSpA, with a particular focus on diagnostic reassessment, treatment modification, and factors associated with changes in diagnosis and treatment after repeat imaging.

## 2. Methods

### 2.1. Study Design and Population

This retrospective, observational, single-center study was conducted at the Rheumatology Clinic of Göztepe Prof. Dr. Süleyman Yalçın City Hospital. Patients evaluated for chronic low back pain, suspected sacroiliitis, or suspected axSpA from January 2010 to January 2026 were retrospectively identified through electronic medical records.

Patients who underwent at least two SIJ MRI examinations were included. The indication for repeat SIJ MRI was determined by the treating rheumatologist according to routine clinical practice. Owing to the retrospective nature of the study, the specific indications for repeat MRI were not systematically documented in the medical records. The cohort primarily included patients undergoing diagnostic evaluation or reassessment for axSpA; however, a subset of patients with previously established axSpA also underwent repeat SIJ MRI as part of therapeutic decision-making. Individuals aged ≥18 years with available clinical follow-up data and accessible MRI reports were eligible for inclusion. Patients with only a single MRI examination, inadequate imaging quality, incomplete clinical data, or alternative conditions affecting the sacroiliac joints, including trauma, infection, or malignancy, were excluded.

### 2.2. Data Collection

Demographic, clinical, laboratory, and imaging data were retrospectively collected from hospital electronic medical records. Recorded variables included age, sex, duration of back pain at presentation, HLA-B27 status, and baseline erythrocyte sedimentation rate (ESR) and C-reactive protein (CRP) levels obtained at the initial rheumatology clinic visit.

Imaging data included the dates of the MRI1 and MRI2 examinations, and the interval between MRI examinations was recorded in months. MRI examinations were performed using routine clinical protocols at our institution. As this was a retrospective study spanning 15 years, imaging protocols were not fully standardized across all examinations. However, most MRI studies included short tau inversion recovery (STIR) or fat-suppressed T2-weighted sequences, together with T1-weighted imaging, obtained in the semicoronal and axial planes.

MRI findings were extracted from official radiology reports generated during routine clinical practice. Because of the retrospective study design, no blinded central re-reading of the original MRI images was performed, including for examinations reported as “suspicious”. This approach reflects real-world MRI interpretation and clinical decision-making. Inflammatory and structural lesions were recorded according to the Assessment of SpondyloArthritis International Society (ASAS) recommendations [4]. Bone marrow edema was classified as an inflammatory lesion, whereas erosions, fat metaplasia, subchondral sclerosis, and ankylosis were recorded as structural lesions. Sacroiliac joint involvement was additionally categorized as unilateral or bilateral.

MRI examinations had originally been reported by radiologists as part of routine clinical practice. For the purposes of this study, existing radiology reports were reviewed by the rheumatologist responsible for the patient’s clinical follow-up, and MRI findings were retrospectively categorized as positive, suspicious, or negative according to the ASAS definition of a positive MRI for sacroiliitis. Positive MRI was defined according to ASAS MRI Working Group recommendations as the presence of active inflammatory lesions highly suggestive of sacroiliitis associated with axSpA, primarily bone marrow edema (BME) located in typical subchondral/periarticular regions [11]. MRI examinations that did not fulfill ASAS-positive criteria but contained equivocal inflammatory findings insufficient for definite sacroiliitis were classified as suspicious.

The clinical diagnosis recorded at the end of follow-up was established retrospectively by the treating rheumatologist based on the overall clinical evaluation during follow-up. This evaluation incorporated clinical features, laboratory findings, imaging results, treatment response, and longitudinal follow-up data, in accordance with current ASAS recommendations and clinical judgment. MRI1 and MRI2 findings were compared to assess the potential contribution of repeat MRI to diagnostic reassessment. Diagnostic reassessment was defined as a change in the treating rheumatologist’s working diagnosis after MRI2 compared with the diagnosis recorded at the time of MRI1 evaluation. This reassessment was based on the integration of repeat MRI findings with longitudinal clinical, laboratory, and follow-up data. The final diagnostic reassessment was recorded retrospectively at the end of follow-up by comparing the working diagnosis documented at the time of MRI1 evaluation with the final working diagnosis established after MRI2 and subsequent clinical follow-up.

### 2.3. Outcome Measures

The primary objective of the study was to evaluate the contribution of repeat SIJ MRI (MRI2) to diagnostic reassessment in patients undergoing evaluation for axSpA. A secondary objective was to assess the impact of MRI2 on treatment modification. Additional objectives included evaluating transitions between MRI1 and MRI2 categories and analyzing longitudinal changes in inflammatory and structural MRI findings over time.

### 2.4. Statistical Analysis

All statistical analyses were performed using Python (version 3.11) with the SciPy, Statsmodels, and Pandas libraries. A two-tailed *p*-value of <0.05 was considered statistically significant. Continuous variables were assessed for normality using the Shapiro–Wilk test. As the majority of continuous variables did not follow a normal distribution, they were expressed as median and interquartile range (IQR). Categorical variables were expressed as frequency and percentage (*n*, %).

For the comparison of MRI1 and MRI2 findings, the Wilcoxon signed-rank test was used for paired ordinal variables (overall MRI result and laterality), and McNemar’s test was applied for paired binary variables (BME, erosion, fat metaplasia, sclerosis, and ankylosis). To identify factors associated with diagnostic reassessment after repeat MRI, patients were divided into two groups: those whose diagnosis changed and those whose diagnosis remained unchanged. Between-group comparisons were performed using the Mann–Whitney U test for continuous variables and Chi-square test or Fisher’s exact test for categorical variables, as appropriate. Variables with *p* < 0.05 in univariate analysis were considered for multivariate modeling. Clinically relevant variables were also included in the binary logistic regression model to identify independent predictors of diagnostic reassessment. Variables were selected based on a combination of univariate statistical significance and predefined clinical relevance rather than automated variable selection procedures. Results were expressed as odds ratios (ORs) with 95% confidence intervals (CIs). An identical analytical approach was applied to identify factors associated with treatment change after repeat MRI. The logistic regression model for treatment change was adjusted for age, sex, HLA-B27 status, CRP, MRI interval, MRI2 overall result, and MRI2 BME positivity. For the transition analysis between MRI1 and MRI2 overall results, a transition matrix was constructed and visualized as a Sankey diagram. Comparisons of MRI interval between outcome groups were illustrated using box plots. Forest plots were generated to display the odds ratios and 95% confidence intervals from both logistic regression models. No imputation was performed for missing data; analyses were conducted on available cases. All tests were two-sided, and statistical significance was set at *p* < 0.05.

### 2.5. Ethics Approval

The study protocol was approved by the Institutional Ethics Committee of Göztepe Prof. Dr. Süleyman Yalçın City Hospital (approval number: 2026/0246). The study was conducted in accordance with the principles of the Declaration of Helsinki.

## 3. Results

A total of 229 patients who underwent repeat SIJ MRI during evaluation for axSpA were included. The median age was 43.0 years (IQR 35.0–50.0), and 169 patients (73.8%) were female. HLA-B27 positivity was detected in 49 patients (21.4%). The median CRP level was 2.2 mg/L (IQR 1.0–7.1), the median ESR was 12.0 mm/h (IQR 6.0–23.0), and the median duration of back pain was 48.0 months (IQR 24.0–96.0). When stratified by sex, the median ESR was significantly higher in female than in male patients (14.0 mm/h [IQR 7.0–25.0] vs. 9.5 mm/h [IQR 3.8–18.8], *p* = 0.022). The median interval between MRI examinations was 34.0 months (IQR 15.0–67.0). Comorbid conditions were present in 47 patients (20.5%), most commonly familial Mediterranean fever (FMF) (*n* = 13), uveitis (*n* = 10), and psoriasis (*n* = 9). Detailed baseline characteristics are presented in Table 1.

**Figure 1:**
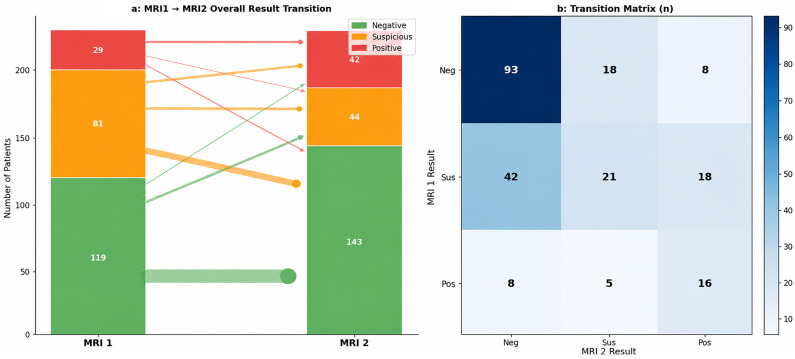
Transition of overall SIJ MRI categories between MRI1 and MRI2. (**a**) Sankey diagram demonstrating transitions between negative, suspicious, and positive MRI categories. (**b**) Transition matrix showing patient numbers for each MRI category change.

On MRI1, 119 patients (52.0%) had negative findings, 81 (35.4%) had suspicious findings, and 29 (12.7%) had positive findings for sacroiliitis. On MRI2, 143 patients (62.4%) were classified as negative, 44 (19.2%) as suspicious, and 42 (18.3%) as positive. Transition analysis of overall MRI categories between MRI1 and MRI2 is shown in Figure 1a, and the corresponding transition matrix is presented in Figure 1b. Among 119 patients with initially negative MRI findings, 8 (6.7%) converted to MRI-positive status on MRI2. Among 81 patients with initially suspicious MRI findings, 18 (22.2%) became MRI-positive on repeat imaging. Conversely, 13 of 29 patients (44.8%) with initially MRI-positive findings demonstrated regression to negative or suspicious findings on MRI2.

**Figure 2:**
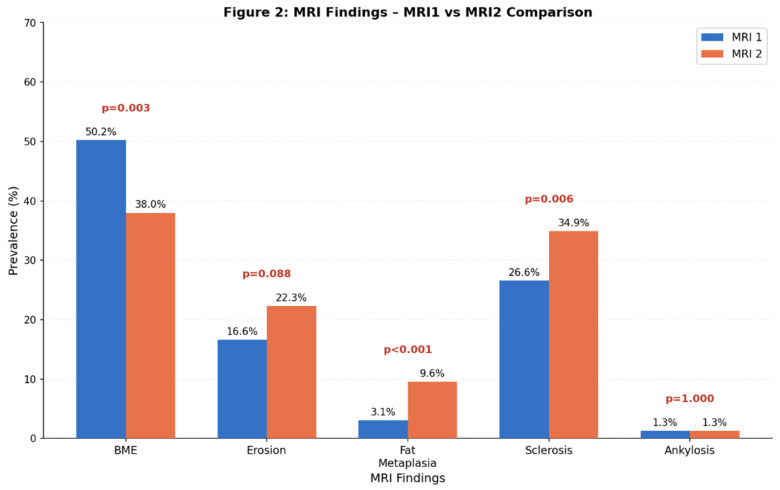
Comparison of inflammatory and structural SIJ MRI findings between MRI1 and MRI2. Bars represent the prevalence of bone marrow edema (BME), erosion, fat metaplasia, sclerosis, and ankylosis on MRI1 and MRI2. *p*-values were calculated using McNemar’s test.

The comparison of inflammatory and structural MRI findings between MRI1 and MRI2 is presented in Table 2 and Figure 2. Bone marrow edema (BME) was significantly less frequent on MRI2 compared with MRI1 (38.0% vs. 50.2%, *p* = 0.003). In contrast, fat metaplasia (9.6% vs. 3.1%, *p* < 0.001) and sclerosis (34.9% vs. 26.6%, *p* = 0.006) were significantly more common on MRI2. No statistically significant differences were observed for erosion (22.3% vs. 16.6%, *p* = 0.088), ankylosis (1.3% vs. 1.3%, *p* = 1.000), overall MRI result score (*p* = 0.383), or laterality of sacroiliac joint involvement (*p* = 0.304).

**Figure 3:**
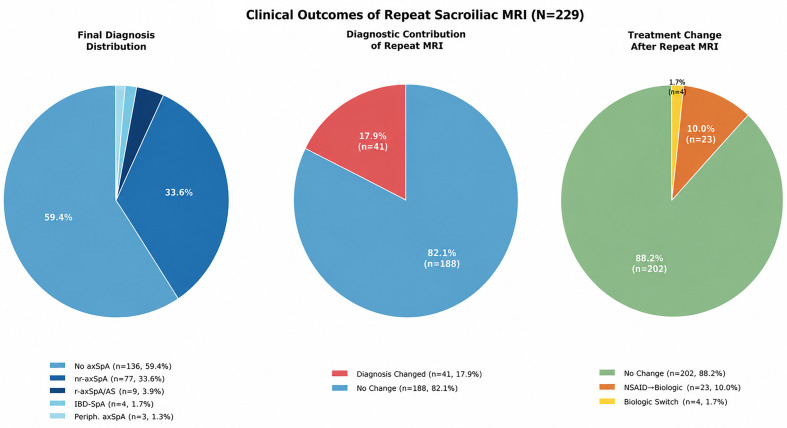
Clinical outcomes associated with repeat sacroiliac joint MRI. The left panel shows final clinical diagnoses, the middle panel shows diagnostic reassessment after repeat MRI, and the right panel demonstrates treatment modifications including escalation from NSAIDs to biologic therapy and biologic switching.

Final clinical diagnoses included no axSpA in 136 patients (59.4%), non-radiographic axSpA in 77 (33.6%), radiographic axSpA/ankylosing spondylitis in 9 (3.9%), IBD-associated SpA in 4 (1.7%), and peripheral SpA in 3 patients (1.3%). Repeat MRI was associated with diagnostic reassessment in 41 patients (17.9%), whereas no diagnostic reassessment occurred in 188 patients (82.1%). Among the 41 patients with diagnostic reassessment, 35 received a new diagnosis of nr-axSpA, 3 were reclassified as radiographic axSpA/ankylosing spondylitis, 2 had their axSpA diagnosis revised to a non-axSpA condition, and 1 was diagnosed with psoriatic arthritis. Treatment modification after repeat MRI occurred in 27 patients (11.8%), including escalation from NSAIDs to biologic therapy in 23 patients (10.0%) and biologic switching in 4 patients (1.7%) (Figure 3). The presence of comorbidity was not significantly associated with diagnostic reassessment after repeat MRI (*p* = 0.67). However, treatment modification after repeat MRI was less frequent in patients with comorbid conditions compared with those without comorbidity (2.1% vs. 14.3%, *p* = 0.02).

**Figure 4:**
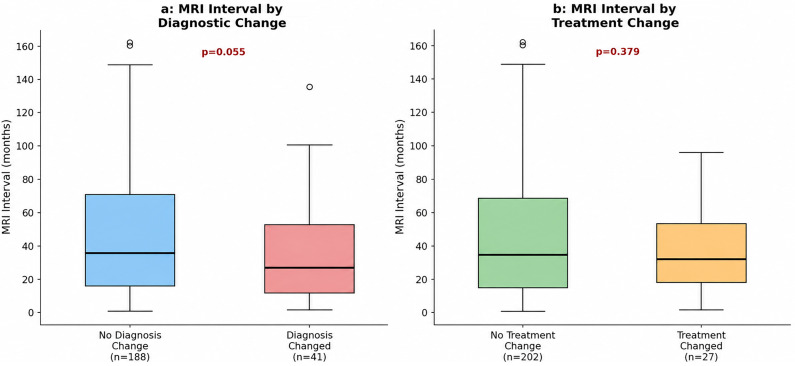
Interval between MRI examinations according to clinical outcomes after repeat MRI. (**a**) MRI interval distribution according to diagnostic change status. (**b**) MRI interval distribution according to treatment modification status. *p*-values were calculated using the Mann–Whitney U test.

In univariate analysis of factors associated with diagnostic reassessment (Table 3a), MRI1 BME positivity was significantly more frequent in patients with diagnostic reassessment compared with those without diagnostic reassessment (65.9% vs. 46.8%, *p* = 0.042). Notably, MRI1-positive findings were observed exclusively among patients without diagnostic reassessment (29/188, 15.4%), whereas none of the 41 patients with diagnostic reassessment had a positive MRI1 finding at baseline (0/41; *p* < 0.001), indicating that patients who were already MRI-positive at baseline virtually never underwent subsequent diagnostic revision. The interval between MRI examinations tended to be shorter in patients with diagnostic reassessment (27.0 vs. 36.0 months, *p* = 0.055) (Figure 4a). No statistically significant differences were identified for age, sex, HLA-B27 positivity, CRP, ESR, or back pain duration. When ESR was analyzed separately by sex, no significant difference was observed between patients with and without diagnostic reassessment in either female (16.5 [11.2–29.2] vs. 12.0 [6.0–22.5], *p* = 0.077) or male patients (6.0 [2.5–11.5] vs. 10.0 [4.0–22.0], *p* = 0.309).

**Figure 5:**
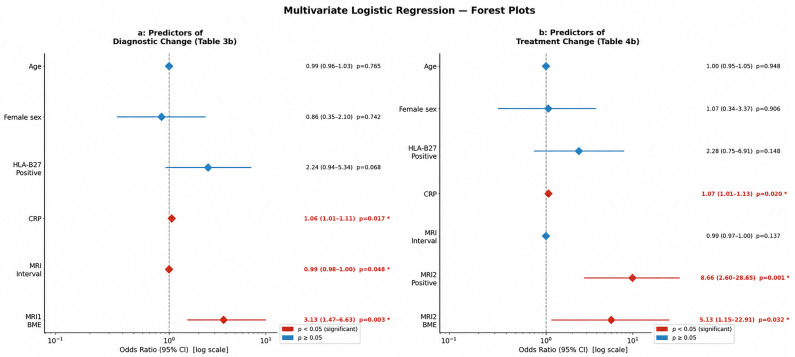
Multivariate logistic regression analyses of factors associated with clinical outcomes after repeat MRI. (**a**) Factors independently associated with diagnostic change. (**b**) Factors independently associated with treatment modification. Odds ratios (ORs) and 95% confidence intervals (CIs) are shown. * *p* < 0.05.

In multivariate logistic regression analysis of diagnostic reassessment (Table 3b), MRI1 BME positivity (OR 3.13, 95% CI 1.47–6.63, *p* = 0.003), higher CRP levels (OR 1.06, 95% CI 1.01–1.11, *p* = 0.017), and shorter MRI interval (OR 0.99, 95% CI 0.98–1.00, *p* = 0.048) were independently associated with diagnostic reassessment after repeat MRI (Figure 5a).

In univariate analysis of factors associated with treatment modification (Table 4a), MRI2-positive findings (74.1% vs. 10.9%, *p* < 0.001), MRI2 BME positivity (85.2% vs. 31.7%, *p* < 0.001), and higher CRP levels (3.7 vs. 2.0 mg/L, *p* = 0.014) were significantly associated with treatment modification. No significant differences were observed for age, sex, HLA-B27 positivity, or ESR. Sex-stratified analysis of ESR similarly showed no significant difference in relation to treatment modification, either in female (17.0 [13.0–33.0] vs. 13.0 [6.0–23.0], *p* = 0.069) or male patients (10.0 [3.8–18.5] vs. 9.0 [4.0–17.5], *p* = 0.944). In addition, no significant difference in MRI interval was observed between patients with and without treatment modification (32.0 vs. 35.0 months, *p* = 0.379) (Figure 4b).

In multivariate logistic regression analysis of treatment modification (Table 4b), MRI2-positive findings (OR 8.66, 95% CI 2.60–28.85, *p* < 0.001), MRI2 BME positivity (OR 5.13, 95% CI 1.15–22.91, *p* = 0.032), and higher CRP levels (OR 1.07, 95% CI 1.01–1.13, *p* = 0.020) remained independently associated with treatment modification after repeat MRI (Figure 5b).

## 4. Discussion

In the present study, repeat SIJ MRI was associated with diagnostic reassessment in 17.9% of patients and treatment modification in 11.8%. Among patients with initially negative or suspicious MRI findings, 13.0% developed MRI positivity on MRI2, whereas 44.8% of those with baseline MRI-positive findings demonstrated regression to negative or suspicious categories over time. Diagnostic reassessment was more frequently observed in patients with baseline BME and higher CRP levels. In addition, BME was less frequent on MRI2, whereas fat metaplasia and sclerosis were more commonly observed on repeat imaging. Taken together, these findings suggest that repeat SIJ MRI may provide additional diagnostic and therapeutic information in selected patients undergoing evaluation for axSpA.

In routine practice, repeat MRI was generally requested in patients with persistent diagnostic uncertainty, ongoing symptoms, or discordance between clinical and imaging findings. Data from longitudinal MRI studies have generally demonstrated low rates of progression to definite sacroiliitis on repeat SIJ MRI following inconclusive baseline examinations [9,12]. Bakker et al. observed that among patients with a negative baseline MRI, only 7.6% developed MRI positivity during follow-up, whereas 38.7% of patients with initially positive MRI findings demonstrated regression to negative MRI status over time [9]. Similarly, Goitein Inbar et al. reported conversion to definite sacroiliitis in only 2.8% of patients on repeat MRI [12]. In our cohort, 13.0% of patients with initially negative or suspicious MRI findings developed MRI positivity on MRI2, whereas 44.8% of initially MRI-positive patients demonstrated regression to negative or suspicious categories over time. These findings further support the dynamic nature of SIJ MRI abnormalities over time. The fluctuating course of axSpA may partly explain the bidirectional MRI changes observed in our cohort [10]. Consistent with the expected evolution of sacroiliac inflammation, active inflammatory lesions such as BME became less frequent on MRI2, whereas structural abnormalities including fat metaplasia and sclerosis became more prominent over time, potentially reflecting progression from active inflammation toward chronic structural lesions. However, because MRI protocols were not standardized, findings were extracted from routine reports without central re-reading, and treatment exposure varied over time, these longitudinal changes cannot be attributed to biological evolution alone and may also reflect protocol variation, interobserver differences, or treatment effects.

Another notable finding of our cohort was the marked predominance of female patients undergoing repeat SIJ MRI evaluation. Although axSpA has historically been considered more common in men [13,14], increasing evidence suggests that diagnosis may be more challenging in women [15]. Female patients with axSpA often present with less typical inflammatory back pain patterns and lower radiographic progression despite substantial disease burden [15,16,17]. Previous studies have also demonstrated longer diagnostic delay, higher disease activity scores, and lower treatment response rates among women with axSpA [15,18,19]. The marked predominance of women in our repeat MRI cohort may reflect greater diagnostic uncertainty encountered in routine clinical practice. Because repeat MRI was generally requested in diagnostically uncertain patients, our cohort likely represents a selected subgroup rather than the overall axSpA population.

Previous studies have suggested that HLA-B27 status may influence the likelihood of MRI conversion during follow-up [9,10]. Bakker et al. reported that the probability of developing MRI-positive sacroiliitis after an initially negative MRI was very low in HLA-B27-negative patients, whereas HLA-B27-positive individuals were more likely to demonstrate MRI conversion over time [9]. Similarly, Sengupta et al. proposed that short-term repeat MRI may be clinically meaningful, particularly in HLA-B27-positive male patients [10]. In our cohort, HLA-B27 positivity was also numerically more common among patients who experienced a diagnostic reassessment after repeat MRI, and multivariate analysis demonstrated a trend toward association, although statistical significance was not reached. Another possible explanation is that the relatively low frequency of HLA-B27 positivity in our cohort may reflect the diagnostic characteristics of patients undergoing repeat MRI evaluation in routine practice. In clinically typical HLA-B27-positive patients, diagnostic confidence may often already be established earlier through the combination of clinical findings and baseline investigations, potentially reducing the need for repeated imaging in some cases. Baseline inflammatory activity may also have influenced the diagnostic contribution of repeat MRI, as MRI1 BME positivity and higher CRP levels were independently associated with diagnostic reassessment after repeat imaging. Although CRP was not significantly associated with diagnostic reassessment in univariate analysis, it emerged as an independent predictor after adjustment for potential confounders in the multivariate model. Furthermore, a shorter interval between MRI examinations was independently associated with diagnostic reassessment. Although the reason for this association cannot be determined from the present study, it may reflect closer follow-up and earlier re-evaluation in patients with greater diagnostic uncertainty. Notably, a positive MRI1 was seen only in patients without diagnostic reassessment, as none of the patients whose diagnosis changed had a positive MRI1. This is a clinically relevant finding in itself, suggesting that a clearly positive baseline MRI already provides diagnostic confidence and reduces the likelihood of later diagnostic revision. Because of this complete separation, the variable could not be retained in the multivariable model. Although penalized methods such as Firth regression can address quasi-complete separation, complete separation still yields unstable estimates with implausibly wide confidence intervals; we therefore report this finding descriptively.

Recent evidence suggests that persistent inflammatory activity on MRI is associated with higher disease activity and may provide clinically relevant information for therapeutic decision-making in axial SpA [7,20]. In addition, real-world data have demonstrated that repeat MRI findings may influence management decisions in selected patients with persistent symptoms despite ongoing therapy [8]. Consistent with these observations, treatment modification in our cohort was more frequently observed in patients with objective evidence of ongoing inflammation, including positive MRI findings, BME on repeat imaging, and higher CRP levels. These findings support an association between inflammatory disease activity and treatment modification following repeat MRI assessment. Although MRI2 overall positivity and MRI2 BME are inherently correlated—as BME constitutes the primary determinant of ASAS-positive MRI classification—both variables were retained in the multivariate model to allow for separate estimation of their independent contributions to treatment modification. MRI2 overall positivity captured the integrated clinical judgment of the treating radiologist, whereas MRI2 BME represented the specific inflammatory lesion driving that classification. The possibility of residual multicollinearity should nonetheless be considered when interpreting the corresponding odds ratios.

The findings of this study should be interpreted in light of several limitations. First, this was a retrospective, single-center study including only patients who underwent repeat MRI, likely representing a diagnostically challenging subgroup with persistent clinical uncertainty. Therefore, the findings may not be fully generalizable to broader axSpA populations. Second, MRI examinations were interpreted within routine clinical practice without central re-reading or consensus assessment, potentially introducing interobserver variability. In addition, the “suspicious” MRI category was derived from radiology report language rather than a standardized scoring system, which may have resulted in variability across radiologists and time points. Third, MRI acquisition protocols and the interval between examinations were not standardized because imaging was performed according to routine clinical practice over a prolonged study period. Furthermore, both the decision to perform repeat MRI and subsequent treatment modifications were made at the discretion of treating physicians, introducing potential indication bias and limiting causal interpretation of the observed associations. Moreover, treatment modification was defined conservatively as escalation from NSAIDs to biologic therapy or biologic switching; other clinically relevant changes such as modifications in NSAID dose or frequency were not captured, and the true rate of treatment modification following repeat MRI may therefore be underestimated. Additionally, diagnostic and therapeutic decisions during follow-up were likely influenced not only by repeat MRI findings but also by the accumulation of clinical and laboratory information over time. Because diagnostic reassessment was defined through the integration of MRI2 with concurrent clinical, laboratory, treatment-response, and follow-up data, the independent contribution of MRI2 could not be isolated from this accumulated information, and the corresponding estimates should be regarded as adjusted associations rather than causal effects. Anti-inflammatory treatment exposure may also have affected longitudinal MRI findings, while detailed data on NSAID use and treatment adherence were not consistently available because of the retrospective study design. Finally, in the multivariate logistic regression model for diagnostic reassessment, MRI1 overall positive result was excluded due to complete separation, which may limit the interpretability of the model. In addition, the relatively small number of treatment modification events compared with the number of covariates included in the multivariable model may have increased the risk of model overfitting and contributed to the wide confidence intervals observed for some estimates. Therefore, these findings should be interpreted with appropriate caution.

The study also has several strengths. To our knowledge, this is among the few real-world studies specifically evaluating the clinical impact of repeat sacroiliac joint MRI in patients undergoing assessment for axial spondyloarthritis. Unlike previous studies that primarily focused on short-term MRI conversion rates, the present study evaluated not only longitudinal imaging changes but also their influence on diagnostic reassessment and treatment decisions. The relatively long follow-up period and the assessment of both inflammatory and structural MRI findings allowed for a more comprehensive evaluation of the dynamic nature of sacroiliac joint abnormalities over time. In addition, the use of longitudinal clinical data enabled the study to better reflect real-world decision-making processes in routine rheumatology practice and enhanced the clinical relevance of the findings.

## 5. Conclusions

Repeat SIJ MRI was associated with diagnostic reassessment and treatment modification in a subset of patients undergoing evaluation for axSpA in routine clinical practice. Although conversion from negative or suspicious MRI findings to definite MRI positivity was relatively uncommon, longitudinal MRI assessment demonstrated that both inflammatory and structural sacroiliac joint abnormalities may evolve over time. Diagnostic reassessment and treatment modification were more frequently observed in patients with evidence of inflammatory disease activity on MRI and elevated inflammatory markers. These findings support the use of repeat MRI as a complementary component of longitudinal clinical assessment rather than as a standalone determinant of diagnosis or treatment decisions. Further prospective studies using standardized MRI protocols and centralized image assessment are needed to better define the role of repeat SIJ MRI in routine practice.

## Figures and Tables

**Table 1:** General Patient Characteristics (*N* = 229).

Characteristic	Total (*N* = 229)
Age at initial rheumatology clinic visit (years), median (IQR)	43.0 (35.0–50.0)
Female sex, *n* (%)	169 (73.8%)
HLA-B27-Positive, *n* (%)	49 (21.4%)
CRP (mg/L), median (IQR)	2.2 (1.0–7.1)
ESR (mm/h), median (IQR)	12.0 (6.0–23.0)
ESR (mm/h), median (IQR), female	14.0 (7.0–25.0)
ESR (mm/h), median (IQR), male	9.5 (3.8–18.8)
Back pain duration (months), median (IQR)	48.0 (24.0–96.0)
MRI interval (months), median (IQR)	34.0 (15.0–67.0)
Comorbidity present, *n* (%)	47 (20.5%)
**MRI1 Overall Result**	
Negative, *n* (%)	119 (52.0%)
Suspicious, *n* (%)	81 (35.4%)
Positive, *n* (%)	29 (12.7%)
**MRI2 Overall Result**	
Negative, *n* (%)	143 (62.4%)
Suspicious, *n* (%)	44 (19.2%)
Positive, *n* (%)	42 (18.3%)
**Final Diagnosis**	
No axSpA, *n* (%)	136 (59.4%)
nr-axSpA, *n* (%)	77 (33.6%)
r-axSpA/AS, *n* (%)	9 (3.9%)
IBD-SpA, *n* (%)	4 (1.7%)
Peripheral SpA, *n* (%)	3 (1.3%)
Diagnosis changed after repeat MRI, *n* (%)	41 (17.9%)
Treatment changed after repeat MRI, *n* (%)	27 (11.8%)

IQR: Interquartile range; HLA: Human leukocyte antigen; CRP: C-reactive protein; ESR: Erythrocyte sedimentation rate; axSpA: Axial spondyloarthritis; nr-axSpA: Non-radiographic axSpA; r-axSpA/AS: Radiographic axSpA/Ankylosing spondylitis; IBD-SpA: Inflammatory bowel disease-associated SpA; MRI: Magnetic resonance imaging.

**Table 2:** Comparison of MRI1 and MRI2 Findings (*N* = 229).

Finding	MRI1	MRI2	*p*-Value
Overall Result, median (IQR)	0.0 (0.0–1.0)	0.0 (0.0–1.0)	0.383
BME, *n* (%)	115 (50.2%)	87 (38.0%)	0.003
Erosion, *n* (%)	38 (16.6%)	51 (22.3%)	0.088
Fat Metaplasia, *n* (%)	7 (3.1%)	22 (9.6%)	<0.001
Sclerosis, *n* (%)	61 (26.6%)	80 (34.9%)	0.006
Ankylosis, *n* (%)	3 (1.3%)	3 (1.3%)	1.000
Laterality, median (IQR)	1.0 (0.0–2.0)	0.0 (0.0–2.0)	0.304

McNemar’s test used for binary findings (BME, erosion, fat metaplasia, sclerosis, ankylosis); Wilcoxon signed-rank test used for ordinal variables (overall result, laterality). MRI1-negative/suspicious → MRI2-positive: 26/200 (13.0%). MRI1-positive → MRI2-negative/suspicious: 13/29 (44.8%). BME: Bone marrow edema. Bold *p*-values indicate statistical significance (*p* < 0.05).

**Table 3:** Factors Associated with Diagnostic Change After Repeat MRI. (**a**) Univariate Analysis. (**b**) Multivariate Logistic Regression.

(**a**)			
**Variable**	**Diagnosis Changed** **(*n* = 41)**	**No Change** **(*n* = 188)**	***p*** **-Value**
Age (years), median (IQR)	40.0 (35.0–49.0)	43.0 (35.0–50.0)	0.836
Female sex, *n* (%)	30 (73.2%)	139 (73.9%)	1.000
HLA-B27-Positive, *n* (%)	13 (31.7%)	36 (19.1%)	0.175
CRP (mg/L), median (IQR)	3.3 (1.0–10.0)	2.0 (1.0–6.3)	0.175
ESR (mm/h), median (IQR)	13.0 (6.0–26.0)	11.0 (5.8–22.2)	0.349
Back pain duration (months)	50.0 (30.0–80.0)	48.0 (24.0–96.0)	0.679
MRI interval (months)	27.0 (12.0–53.0)	36.0 (16.0–71.2)	0.055
MRI1 Overall Positive, *n* (%)	0 (0.0%)	29 (15.4%)	<0.001
MRI1 BME Positive, *n* (%)	27 (65.9%)	88 (46.8%)	0.042
(**b**)			
**Variable**	**OR**	**95% CI**	***p*** **-Value**
Age	0.99	0.96–1.03	0.765
Female sex	0.86	0.35–2.10	0.742
HLA-B27-positive	2.24	0.94–5.34	0.068
CRP	1.06	1.01–1.11	0.017
MRI interval	0.99	0.98–1.00	0.048
MRI1 BME	3.13	1.47–6.63	0.003

OR: Odds ratio; CI: Confidence interval. Mann–Whitney U test for continuous variables; Chi-square or Fisher’s exact test for categorical variables. Logistic regression adjusted for age, sex, HLA-B27, CRP, MRI interval, MRI1 overall result, and MRI1 BME. MRI1 overall positive result was significant in univariate analysis but could not be retained in the multivariate model because no patient with diagnostic reassessment had a positive MRI1 result, resulting in complete separation.

**Table 4:** Factors Associated with Treatment Change After Repeat MRI. (**a**) Univariate Analysis. (**b**) Multivariate Logistic Regression.

(**a**)			
**Variable**	**Treatment Changed** **(*n* = 27)**	**No Change** **(*n* = 202)**	***p*** **-Value**
Age (years), median (IQR)	38.0 (34.5–47.5)	44.0 (35.0–50.0)	0.322
Female sex, *n* (%)	17 (63.0%)	152 (75.2%)	0.258
HLA-B27-Positive, *n* (%)	10 (37.0%)	39 (19.3%)	0.101
CRP (mg/L), median (IQR)	3.7 (1.8–15.0)	2.0 (0.9–6.6)	0.014
ESR (mm/h), median (IQR)	16.0 (9.5–27.5)	11.5 (5.2–22.8)	0.219
MRI interval (months)	32.0 (18.5–53.5)	35.0 (15.0–68.8)	0.379
MRI2 Overall Positive, *n* (%)	20 (74.1%)	22 (10.9%)	<0.001
MRI2 BME Positive, *n* (%)	23 (85.2%)	64 (31.7%)	<0.001
(**b**)			
**Variable**	**OR**	**95% CI**	***p*** **-Value**
Age	1.00	0.95–1.05	0.948
Female sex	1.07	0.34–3.37	0.906
HLA-B27-positive	2.28	0.75–6.93	0.148
CRP	1.07	1.01–1.13	0.020
MRI interval	0.99	0.97–1.00	0.137
MRI2 Positive	8.66	2.60–28.85	<0.001
MRI2 BME	5.13	1.15–22.91	0.032

OR: Odds ratio; CI: Confidence interval. Mann–Whitney U test for continuous variables; Chi-square or Fisher’s exact test for categorical variables. Logistic regression adjusted for age, sex, HLA-B27, CRP, MRI interval, MRI2 overall result, and MRI2 BME.

## Data Availability

The data presented in this study are available from the corresponding author upon reasonable request. The data are not publicly available due to privacy and ethical restrictions.
